# 6-[(2,4-Di­methyl­anilino)methyl­idene]-2-hy­droxy­cyclo­hexa-2,4-dienone

**DOI:** 10.1107/S1600536813012233

**Published:** 2013-05-11

**Authors:** Shaukat Shujah, Saqib Ali, Zia-ur-Rehman, M. Nawaz Tahir, Auke Meetsma

**Affiliations:** a Department of Chemistry, Kohat University of Science & Technology, Kohat 26000, Kohat, Pakistan; b Department of Chemistry, Quaid-i-Azam University, Islamabad, Pakistan; c Department of Physics, University of Sargodha, Sargodha, Pakistan; d Crystal Structure Center, Chemical Physics, Zernike Institute for Advanced Materials, University of Groningen, Nijenborgh 4, NL-9747 AG Groningen, The Netherlands

## Abstract

In the title compound, C_15_H_15_NO_2_, the dihedral angle between the aromatic rings is 5.86 (6)°, and an intra­molecular N—H⋯O hydrogen bond generates an *S*(6) motif, which helps to stabilize the enamine–keto tautomer. An intra­molecular O—H⋯O hydrogen bond also occurs. In the crystal, inversion dimers linked by pairs of O—H⋯O hydrogen bonds generate *R*_2_^2^(10) loops. A C—H⋯O inter­action links the dimers into [010] chains and aromatic π–π stacking [centroid–centroid separation = 3.6131 (9) Å] also occurs.

## Related literature

For a related structure and background to Schiff bases, see: Shuja *et al.* (2007 ▶). For further structural aspects, see: Blagus & Kaitner (2011 ▶).
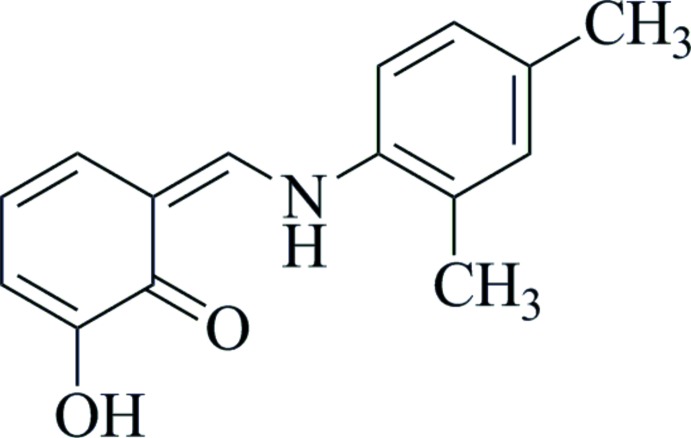


## Experimental

### 

#### Crystal data

C_15_H_15_NO_2_
*M* *_r_* = 241.28
Triclinic, 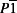

*a* = 7.6731 (8) Å
*b* = 8.4348 (9) Å
*c* = 10.5806 (12) Å
α = 80.6107 (18)°
β = 75.8216 (19)°
γ = 63.3573 (16)°
*V* = 592.31 (11) Å^3^
*Z* = 2
Mo *K*α radiation
μ = 0.09 mm^−1^
*T* = 100 K
0.53 × 0.48 × 0.29 mm

#### Data collection

Bruker SMART APEX CCD area-detector diffractometer
Absorption correction: multi-scan (*SADABS*; Bruker, 2006 ▶) *T*_min_ = 0.943, *T*_max_ = 0.974
4681 measured reflections
2363 independent reflections
2148 reflections with *I* > 2σ(*I*)
*R*_int_ = 0.009

#### Refinement

*R*[*F* ^2^ > 2σ(*F* ^2^)] = 0.043
*wR*(*F* ^2^) = 0.123
*S* = 1.09
2363 reflections
223 parameters
All H-atom parameters refined
Δρ_max_ = 0.29 e Å^−3^
Δρ_min_ = −0.27 e Å^−3^

### 

Data collection: *SMART* (Bruker, 2006 ▶); cell refinement: *SAINT-Plus* (Bruker, 2006 ▶); data reduction: *SAINT-Plus*; program(s) used to solve structure: *SIR2004* (Burla *et al.*, 2005 ▶); program(s) used to refine structure: *SHELXL97* (Sheldrick, 2008 ▶); molecular graphics: *PLUTO* (Meetsma, 2006 ▶) and *PLATON* (Spek, 2009 ▶); software used to prepare material for publication: *PLATON*.

## Supplementary Material

Crystal structure: contains datablock(s) global, I. DOI: 10.1107/S1600536813012233/hb7078sup1.cif

Structure factors: contains datablock(s) I. DOI: 10.1107/S1600536813012233/hb7078Isup2.hkl

Supplementary material file. DOI: 10.1107/S1600536813012233/hb7078Isup3.cml

Additional supplementary materials:  crystallographic information; 3D view; checkCIF report

## Figures and Tables

**Table 1:** Hydrogen-bond geometry (Å, °)

*D*—H⋯*A*	*D*—H	H⋯*A*	*D*⋯*A*	*D*—H⋯*A*
O1—H21⋯O2	0.85 (2)	2.340 (17)	2.7674 (12)	111.3 (12)
O1—H21⋯O2^i^	0.85 (2)	2.00 (2)	2.7320 (13)	143.4 (15)
N1—H31⋯O2	1.00 (2)	1.72 (2)	2.5873 (12)	142.7 (18)
C13—H13⋯O1^ii^	0.980 (14)	2.557 (14)	3.3069 (14)	133.3 (13)

Symmetry codes: (i) 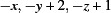; (ii) 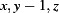.

**Fig. 1.**
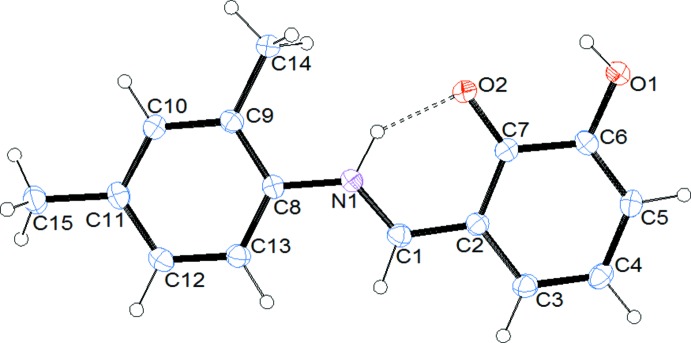
The molecular stucture of (I), with displacement ellipsoids drawn at the 50% probability level. The intramolecular N—H···O hydrogen bond is shown as dashed lines.

**Fig. 2.**
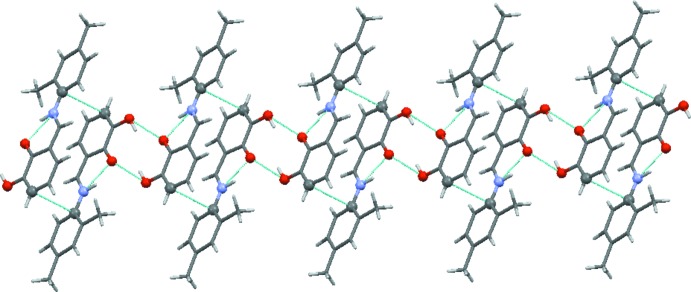
Schiff base molecules connected by π–π interactions and O—H···O hydrogen bonds.

## supplementary crystallographic information

### Comment

As part of our ongoing studies of Schiff bases (Shuja *et al.* 2007), here we report the synthesis and structure of title compound, (I), (Fig. 1). In the molecular structure of (I) the bond distances C7–O2 [1.298 (13) Å] and C1–N1 [1.311 (14) Å] indicate keto-amino tautomeric form. This is further confirmed by a formation of strong intramolecular hydrogen bond N–H···O [N···O = 2.587 (12) Å] resulting in an S(6) ring. The C8–N1–C1–C2 torsion angle is 179.91 (12). The C1–N1 bond length [1.311 (14) Å] is smaller than the N1–C8 bond length [1.417 (14) Å]. The C1–C2 bond length [1.416 (16) Å] indicate a double-bond character and the short C5–C6 bond distance [1.372 (16) Å] of benzene core suggests the presence of quinoid effect (Blagus *et al.*, 2011). The molecules are connected by π–π interactions and O–H···O hydrogen bonds forming a two dimensional network (Fig. 2)

### Experimental

An ethanolic solution (50 ml) of 2,4-dimethylaniline (2.5 mmol, 0.30 g) was added dropwise with constant stirring to a hot ethanolic solution (50 ml) of 2,3-dihydroxybenzaldehyde (2.5 mmol, 0.34 g) in a round bottomed flask equipped with a water condenser. The reaction mixture was kept under reflux for 2 h, cooled and kept at room temperature for 48 h. Red blocks of (I) were obtained on slow evaporation of the solvent.

### Crystal data

**Table:**

C_15_H_15_NO_2_	*Z* = 2
*M**_r_* = 241.28	*F*(000) = 256
Triclinic, *P*1	The final unit cell was obtained from the xyz centroids of 3645 reflections after integration using the SAINTPLUS software package (Bruker, 2000).
Hall symbol: -P 1	*D*_x_ = 1.353 Mg m^−^^3^
*a* = 7.6731 (8) Å	Mo *K*α radiation, λ = 0.71073 Å
*b* = 8.4348 (9) Å	Cell parameters from 3645 reflections
*c* = 10.5806 (12) Å	θ = 2.7–29.6°
α = 80.6107 (18)°	µ = 0.09 mm^−^^1^
β = 75.8216 (19)°	*T* = 100 K
γ = 63.3573 (16)°	Block, red
*V* = 592.31 (11) Å^3^	0.53 × 0.48 × 0.29 mm

### Data collection

**Table:**

Bruker SMART APEX CCD area-detector diffractometer	2363 independent reflections
Radiation source: fine focus sealed Siemens Mo tube	2148 reflections with *I* > 2σ(*I*)
Parallel mounted graphite monochromator	*R*_int_ = 0.009
Detector resolution: 4096x4096 / 62x62 (binned 512) pixels mm^-1^	θ_max_ = 26.4°, θ_min_ = 2.7°
φ and ω scans	*h* = −9→9
Absorption correction: multi-scan (*SADABS*; Bruker, 2006)	*k* = −10→10
*T*_min_ = 0.943, *T*_max_ = 0.974	*l* = −13→13
4681 measured reflections	

### Refinement

**Table:**

Refinement on *F*^2^	Primary atom site location: structure-invariant direct methods
Least-squares matrix: full	Secondary atom site location: structure-invariant direct methods
*R*[*F*^2^ > 2σ(*F*^2^)] = 0.043	Hydrogen site location: inferred from neighbouring sites
*wR*(*F*^2^) = 0.123	All H-atom parameters refined
*S* = 1.09	*w* = 1/[σ^2^(*F*_o_^2^) + (0.0825*P*)^2^ + 0.0862*P*] where *P* = (*F*_o_^2^ + 2*F*_c_^2^)/3
2363 reflections	(Δ/σ)_max_ < 0.001
223 parameters	Δρ_max_ = 0.29 e Å^−^^3^
0 restraints	Δρ_min_ = −0.27 e Å^−^^3^

### Special details

**Table:**

Geometry. Bond distances, angles *etc*. have been calculated using the rounded fractional coordinates. All su's are estimated from the variances of the (full) variance-covariance matrix. The cell e.s.d.'s are taken into account in the estimation of distances, angles and torsion angles
Refinement. Refinement of *F*^2^ against ALL reflections. The weighted *R*-factor *wR* and goodness of fit *S* are based on *F*^2^, conventional *R*-factors *R* are based on *F*, with *F* set to zero for negative *F*^2^. The threshold expression of *F*^2^ > σ(*F*^2^) is used only for calculating *R*-factors(gt) *etc*. and is not relevant to the choice of reflections for refinement. *R*-factors based on *F*^2^ are statistically about twice as large as those based on *F*, and *R*- factors based on ALL data will be even larger.

### Fractional atomic coordinates and isotropic or equivalent isotropic displacement parameters (Å^2^)

**Table:**

	*x*	*y*	*z*	*U*_iso_*/*U*_eq_	
O1	0.14548 (13)	0.99696 (10)	0.64979 (8)	0.0261 (3)	
O2	0.14914 (11)	0.77675 (9)	0.48083 (7)	0.0213 (2)	
N1	0.23552 (13)	0.45820 (12)	0.42735 (8)	0.0179 (3)	
C1	0.29515 (15)	0.40370 (14)	0.53911 (10)	0.0198 (3)	
C2	0.28631 (15)	0.52113 (14)	0.62454 (10)	0.0180 (3)	
C3	0.35444 (16)	0.45246 (14)	0.74426 (10)	0.0214 (3)	
C4	0.35457 (16)	0.56226 (14)	0.82597 (10)	0.0217 (3)	
C5	0.28454 (16)	0.74722 (14)	0.79198 (10)	0.0209 (3)	
C6	0.21286 (16)	0.81822 (14)	0.67902 (10)	0.0195 (3)	
C7	0.21212 (15)	0.70814 (14)	0.58908 (10)	0.0178 (3)	
C8	0.23908 (15)	0.35066 (13)	0.33599 (10)	0.0173 (3)	
C9	0.18384 (15)	0.43224 (13)	0.21664 (10)	0.0182 (3)	
C10	0.18940 (16)	0.32622 (14)	0.12556 (10)	0.0194 (3)	
C11	0.24356 (16)	0.14426 (14)	0.15117 (10)	0.0203 (3)	
C12	0.29389 (16)	0.06799 (14)	0.27204 (11)	0.0215 (3)	
C13	0.29397 (16)	0.16855 (14)	0.36332 (10)	0.0201 (3)	
C14	0.11771 (17)	0.62921 (14)	0.18686 (10)	0.0220 (3)	
C15	0.24119 (18)	0.03446 (15)	0.05288 (12)	0.0250 (3)	
H1	0.351 (2)	0.273 (2)	0.5651 (14)	0.034 (4)*	
H3	0.400 (2)	0.323 (2)	0.7672 (15)	0.042 (4)*	
H4	0.401 (2)	0.5179 (18)	0.9075 (14)	0.027 (3)*	
H5	0.286 (2)	0.8279 (19)	0.8468 (14)	0.031 (4)*	
H10	0.1540 (19)	0.3814 (17)	0.0398 (13)	0.024 (3)*	
H12	0.326 (2)	−0.062 (2)	0.2955 (14)	0.036 (4)*	
H13	0.328 (2)	0.1118 (18)	0.4479 (13)	0.026 (3)*	
H14	0.218 (2)	0.6672 (19)	0.1967 (14)	0.037 (4)*	
H14'	0.096 (2)	0.6606 (19)	0.0969 (14)	0.033 (4)*	
H14"	−0.010 (2)	0.6970 (19)	0.2456 (14)	0.033 (4)*	
H15	0.251 (3)	0.088 (2)	−0.0373 (18)	0.049 (5)*	
H15'	0.353 (2)	−0.084 (2)	0.0470 (16)	0.047 (4)*	
H15"	0.122 (3)	0.016 (2)	0.0749 (16)	0.046 (4)*	
H21	0.076 (3)	1.025 (2)	0.5909 (19)	0.052 (5)*	
H31	0.187 (3)	0.590 (3)	0.412 (2)	0.088 (7)*	

### Atomic displacement parameters (Å^2^)

**Table:**

	*U*^11^	*U*^22^	*U*^33^	*U*^12^	*U*^13^	*U*^23^
O1	0.0360 (5)	0.0169 (4)	0.0268 (4)	−0.0088 (3)	−0.0143 (4)	−0.0005 (3)
O2	0.0259 (4)	0.0177 (4)	0.0188 (4)	−0.0070 (3)	−0.0078 (3)	0.0007 (3)
N1	0.0187 (5)	0.0161 (4)	0.0183 (4)	−0.0068 (3)	−0.0035 (3)	−0.0018 (3)
C1	0.0189 (5)	0.0172 (5)	0.0211 (5)	−0.0060 (4)	−0.0037 (4)	−0.0011 (4)
C2	0.0169 (5)	0.0176 (5)	0.0178 (5)	−0.0058 (4)	−0.0031 (4)	−0.0015 (4)
C3	0.0210 (5)	0.0194 (5)	0.0207 (5)	−0.0057 (4)	−0.0053 (4)	−0.0002 (4)
C4	0.0188 (5)	0.0251 (5)	0.0185 (5)	−0.0061 (4)	−0.0057 (4)	−0.0009 (4)
C5	0.0201 (5)	0.0239 (5)	0.0191 (5)	−0.0086 (4)	−0.0034 (4)	−0.0051 (4)
C6	0.0190 (5)	0.0173 (5)	0.0211 (5)	−0.0069 (4)	−0.0028 (4)	−0.0028 (4)
C7	0.0157 (5)	0.0193 (5)	0.0169 (5)	−0.0066 (4)	−0.0026 (4)	−0.0009 (4)
C8	0.0156 (5)	0.0175 (5)	0.0188 (5)	−0.0070 (4)	−0.0025 (4)	−0.0029 (4)
C9	0.0167 (5)	0.0170 (5)	0.0200 (5)	−0.0067 (4)	−0.0030 (4)	−0.0014 (4)
C10	0.0192 (5)	0.0206 (5)	0.0187 (5)	−0.0083 (4)	−0.0043 (4)	−0.0014 (4)
C11	0.0180 (5)	0.0200 (5)	0.0233 (5)	−0.0081 (4)	−0.0021 (4)	−0.0055 (4)
C12	0.0206 (5)	0.0169 (5)	0.0259 (5)	−0.0073 (4)	−0.0045 (4)	−0.0010 (4)
C13	0.0204 (5)	0.0177 (5)	0.0215 (5)	−0.0074 (4)	−0.0053 (4)	0.0001 (4)
C14	0.0283 (6)	0.0172 (5)	0.0204 (5)	−0.0084 (4)	−0.0083 (4)	0.0002 (4)
C15	0.0275 (6)	0.0221 (5)	0.0272 (6)	−0.0102 (5)	−0.0064 (4)	−0.0061 (4)

### Geometric parameters (Å, º)

**Table:**

O1—C6	1.3649 (13)	C10—C11	1.3961 (15)
O2—C7	1.2978 (13)	C11—C12	1.3961 (16)
O1—H21	0.85 (2)	C11—C15	1.5094 (17)
N1—C1	1.3107 (14)	C12—C13	1.3857 (16)
N1—C8	1.4174 (14)	C1—H1	1.005 (15)
N1—H31	1.00 (2)	C3—H3	0.993 (15)
C1—C2	1.4158 (16)	C4—H4	0.968 (15)
C2—C3	1.4237 (15)	C5—H5	0.969 (15)
C2—C7	1.4350 (15)	C10—H10	0.992 (14)
C3—C4	1.3666 (16)	C12—H12	1.012 (15)
C4—C5	1.4184 (15)	C13—H13	0.980 (14)
C5—C6	1.3721 (16)	C14—H14	0.988 (17)
C6—C7	1.4365 (16)	C14—H14'	0.980 (15)
C8—C9	1.4018 (15)	C14—H14"	0.994 (15)
C8—C13	1.3982 (15)	C15—H15	0.987 (18)
C9—C14	1.5061 (15)	C15—H15'	0.982 (16)
C9—C10	1.3989 (15)	C15—H15"	0.96 (2)
			
O1···O2	2.7674 (12)	C4···H14^iv^	2.938 (16)
O1···C13^i^	3.3069 (14)	C4···H14'^vi^	3.059 (15)
O1···O2^ii^	2.7320 (13)	C5···H15"^iii^	2.98 (2)
O1···C14^ii^	3.3713 (14)	C7···H31	2.35 (2)
O2···O1^ii^	2.7320 (13)	C12···H3^vii^	3.096 (15)
O2···C12^i^	3.4092 (14)	C13···H21^v^	2.99 (2)
O2···O1	2.7674 (12)	C13···H1	2.635 (15)
O2···N1	2.5873 (12)	C14···H31	2.50 (2)
O2···C13^iii^	3.2538 (16)	C15···H5^viii^	2.871 (15)
O1···H13^i^	2.557 (14)	H1···C13	2.635 (15)
O1···H14"^ii^	2.633 (14)	H1···H3	2.39 (2)
O2···H31	1.72 (2)	H1···H13	2.07 (2)
O2···H12^i^	2.649 (16)	H3···H1	2.39 (2)
O2···H21	2.340 (17)	H3···C12^vii^	3.096 (15)
O2···H21^ii^	1.999 (19)	H3···H12^vii^	2.31 (2)
N1···O2	2.5873 (12)	H5···C15^ix^	2.871 (15)
N1···H14	2.762 (14)	H5···H15^ix^	2.57 (2)
N1···H14"	2.900 (15)	H5···H15'^ix^	2.59 (2)
C1···C2^iv^	3.5296 (18)	H10···C4^x^	3.015 (14)
C1···C7^iv^	3.4250 (18)	H10···H14'	2.35 (2)
C2···C9^iii^	3.4592 (18)	H10···H15	2.46 (2)
C2···C1^iv^	3.5296 (18)	H10···H10^xi^	2.55 (2)
C2···C2^iv^	3.5848 (17)	H12···O2^v^	2.649 (16)
C4···C9^iv^	3.4797 (19)	H12···H3^vii^	2.31 (2)
C4···C8^iv^	3.4962 (18)	H13···O1^v^	2.557 (14)
C5···C13^iv^	3.5694 (19)	H13···C1	2.675 (14)
C5···C8^iv^	3.3332 (18)	H13···H1	2.07 (2)
C6···C12^iii^	3.4839 (19)	H13···H21^v^	2.46 (3)
C6···C11^iii^	3.4209 (19)	H14···N1	2.762 (14)
C7···C12^iii^	3.5121 (18)	H14···H31	2.25 (3)
C7···C13^iii^	3.4649 (19)	H14···C4^iv^	2.938 (16)
C7···C1^iv^	3.4250 (18)	H14'···C4^x^	3.059 (15)
C7···C8^iii^	3.5955 (18)	H14'···H10	2.35 (2)
C8···C5^iv^	3.3332 (18)	H14"···N1	2.900 (15)
C8···C4^iv^	3.4962 (18)	H14"···H31	2.40 (3)
C8···C7^iii^	3.5955 (18)	H14"···O1^ii^	2.633 (14)
C9···C2^iii^	3.4592 (18)	H14"···C1^iii^	3.048 (16)
C9···C4^iv^	3.4797 (19)	H15···H5^viii^	2.57 (2)
C11···C6^iii^	3.4209 (19)	H15···H10	2.46 (2)
C12···C7^iii^	3.5121 (18)	H15'···H5^viii^	2.59 (2)
C12···O2^v^	3.4092 (14)	H15"···C5^iii^	2.98 (2)
C12···C6^iii^	3.4839 (19)	H21···O2	2.340 (17)
C13···C5^iv^	3.5694 (19)	H21···C13^i^	2.99 (2)
C13···C7^iii^	3.4649 (19)	H21···H13^i^	2.46 (3)
C13···O2^iii^	3.2538 (16)	H21···O2^ii^	2.00 (2)
C13···O1^v^	3.3069 (14)	H31···O2	1.72 (2)
C14···O1^ii^	3.3713 (14)	H31···C7	2.35 (2)
C1···H14"^iii^	3.048 (16)	H31···C14	2.50 (2)
C1···H13	2.675 (14)	H31···H14	2.25 (3)
C4···H10^vi^	3.015 (14)	H31···H14"	2.40 (3)
			
C6—O1—H21	108.2 (11)	C8—C13—C12	119.90 (10)
C1—N1—C8	126.62 (9)	N1—C1—H1	118.8 (9)
C1—N1—H31	110.9 (13)	C2—C1—H1	118.4 (9)
C8—N1—H31	122.5 (12)	C2—C3—H3	118.1 (9)
N1—C1—C2	122.78 (10)	C4—C3—H3	121.0 (9)
C1—C2—C3	119.73 (10)	C3—C4—H4	122.0 (8)
C1—C2—C7	119.92 (10)	C5—C4—H4	118.2 (8)
C3—C2—C7	120.35 (10)	C4—C5—H5	121.7 (9)
C2—C3—C4	120.82 (10)	C6—C5—H5	117.5 (9)
C3—C4—C5	119.78 (10)	C9—C10—H10	119.3 (8)
C4—C5—C6	120.82 (10)	C11—C10—H10	118.6 (8)
O1—C6—C5	119.25 (10)	C11—C12—H12	119.8 (9)
O1—C6—C7	119.23 (10)	C13—C12—H12	118.9 (9)
C5—C6—C7	121.50 (10)	C8—C13—H13	120.6 (8)
C2—C7—C6	116.70 (10)	C12—C13—H13	119.5 (8)
O2—C7—C2	122.55 (10)	C9—C14—H14	111.6 (8)
O2—C7—C6	120.74 (9)	C9—C14—H14'	109.8 (9)
N1—C8—C13	121.55 (9)	C9—C14—H14"	110.7 (8)
N1—C8—C9	118.11 (9)	H14—C14—H14'	108.3 (13)
C9—C8—C13	120.34 (10)	H14—C14—H14"	108.7 (13)
C8—C9—C10	118.30 (9)	H14'—C14—H14"	107.6 (13)
C8—C9—C14	121.27 (9)	C11—C15—H15	113.3 (11)
C10—C9—C14	120.43 (9)	C11—C15—H15'	113.3 (10)
C9—C10—C11	122.16 (10)	C11—C15—H15"	111.1 (10)
C10—C11—C15	120.97 (10)	H15—C15—H15'	104.2 (14)
C10—C11—C12	118.03 (10)	H15—C15—H15"	107.9 (18)
C12—C11—C15	120.97 (10)	H15'—C15—H15"	106.4 (14)
C11—C12—C13	121.25 (10)		
			
C8—N1—C1—C2	179.91 (12)	O1—C6—C7—C2	179.47 (11)
C1—N1—C8—C9	−174.84 (12)	C5—C6—C7—O2	−177.99 (12)
C1—N1—C8—C13	5.77 (19)	C5—C6—C7—C2	1.19 (18)
N1—C1—C2—C3	−179.77 (12)	N1—C8—C9—C10	179.26 (11)
N1—C1—C2—C7	−0.87 (19)	N1—C8—C9—C14	−1.44 (18)
C1—C2—C3—C4	177.46 (12)	C13—C8—C9—C10	−1.34 (18)
C7—C2—C3—C4	−1.44 (19)	C13—C8—C9—C14	177.96 (12)
C1—C2—C7—O2	0.84 (19)	N1—C8—C13—C12	179.40 (12)
C1—C2—C7—C6	−178.32 (11)	C9—C8—C13—C12	0.0 (2)
C3—C2—C7—O2	179.74 (12)	C8—C9—C10—C11	1.45 (19)
C3—C2—C7—C6	0.58 (18)	C14—C9—C10—C11	−177.87 (12)
C2—C3—C4—C5	0.53 (19)	C9—C10—C11—C12	−0.20 (19)
C3—C4—C5—C6	1.26 (19)	C9—C10—C11—C15	177.81 (12)
C4—C5—C6—O1	179.58 (12)	C10—C11—C12—C13	−1.2 (2)
C4—C5—C6—C7	−2.1 (2)	C15—C11—C12—C13	−179.20 (12)
O1—C6—C7—O2	0.29 (18)	C11—C12—C13—C8	1.3 (2)

Symmetry codes: (i) *x*, *y*+1, *z*; (ii) −*x*, −*y*+2, −*z*+1; (iii) −*x*, −*y*+1, −*z*+1; (iv) −*x*+1, −*y*+1, −*z*+1; (v) *x*, *y*−1, *z*; (vi) *x*, *y*, *z*+1; (vii) −*x*+1, −*y*, −*z*+1; (viii) *x*, *y*−1, *z*−1; (ix) *x*, *y*+1, *z*+1; (x) *x*, *y*, *z*−1; (xi) −*x*, −*y*+1, −*z*.

### Hydrogen-bond geometry (Å, º)

**Table:**

*D*—H···*A*	*D*—H	H···*A*	*D*···*A*	*D*—H···*A*
O1—H21···O2	0.85 (2)	2.340 (17)	2.7674 (12)	111.3 (12)
O1—H21···O2^ii^	0.85 (2)	2.00 (2)	2.7320 (13)	143.4 (15)
N1—H31···O2	1.00 (2)	1.72 (2)	2.5873 (12)	142.7 (18)
C13—H13···O1^v^	0.980 (14)	2.557 (14)	3.3069 (14)	133.3 (13)

Symmetry codes: (ii) −*x*, −*y*+2, −*z*+1; (v) *x*, *y*−1, *z*.
